# DiSignAtlas: an atlas of human and mouse disease signatures based on bulk and single-cell transcriptomics

**DOI:** 10.1093/nar/gkad961

**Published:** 2023-11-01

**Authors:** Zhaoyu Zhai, Zhewei Lin, Xuehang Meng, Xiao Zheng, Yujia Du, Zhi Li, Xuelu Zhang, Chang Liu, Lu Zhou, Xu Zhang, Zhihao Tian, Qinfeng Ma, Jinhao Li, Qiang Li, Jianbo Pan

**Affiliations:** Precision Medicine Center, The Second Affiliated Hospital of Chongqing Medical University, Chongqing 400010, China; Basic Medicine Research and Innovation Center for Novel Target and Therapeutic Intervention, Ministry of Education, Institute of Life Sciences, Chongqing Medical University, Chongqing 400016, China; Basic Medicine Research and Innovation Center for Novel Target and Therapeutic Intervention, Ministry of Education, Institute of Life Sciences, Chongqing Medical University, Chongqing 400016, China; Basic Medicine Research and Innovation Center for Novel Target and Therapeutic Intervention, Ministry of Education, Institute of Life Sciences, Chongqing Medical University, Chongqing 400016, China; Basic Medicine Research and Innovation Center for Novel Target and Therapeutic Intervention, Ministry of Education, Institute of Life Sciences, Chongqing Medical University, Chongqing 400016, China; Basic Medicine Research and Innovation Center for Novel Target and Therapeutic Intervention, Ministry of Education, Institute of Life Sciences, Chongqing Medical University, Chongqing 400016, China; Basic Medicine Research and Innovation Center for Novel Target and Therapeutic Intervention, Ministry of Education, Institute of Life Sciences, Chongqing Medical University, Chongqing 400016, China; Basic Medicine Research and Innovation Center for Novel Target and Therapeutic Intervention, Ministry of Education, Institute of Life Sciences, Chongqing Medical University, Chongqing 400016, China; Basic Medicine Research and Innovation Center for Novel Target and Therapeutic Intervention, Ministry of Education, Institute of Life Sciences, Chongqing Medical University, Chongqing 400016, China; Basic Medicine Research and Innovation Center for Novel Target and Therapeutic Intervention, Ministry of Education, Institute of Life Sciences, Chongqing Medical University, Chongqing 400016, China; Basic Medicine Research and Innovation Center for Novel Target and Therapeutic Intervention, Ministry of Education, Institute of Life Sciences, Chongqing Medical University, Chongqing 400016, China; Basic Medicine Research and Innovation Center for Novel Target and Therapeutic Intervention, Ministry of Education, Institute of Life Sciences, Chongqing Medical University, Chongqing 400016, China; Basic Medicine Research and Innovation Center for Novel Target and Therapeutic Intervention, Ministry of Education, Institute of Life Sciences, Chongqing Medical University, Chongqing 400016, China; Hepatobiliary Surgery, The Second Affiliated Hospital of Chongqing Medical University, Chongqing 400010, China; Basic Medicine Research and Innovation Center for Novel Target and Therapeutic Intervention, Ministry of Education, Institute of Life Sciences, Chongqing Medical University, Chongqing 400016, China; Precision Medicine Center, The Second Affiliated Hospital of Chongqing Medical University, Chongqing 400010, China; Basic Medicine Research and Innovation Center for Novel Target and Therapeutic Intervention, Ministry of Education, Institute of Life Sciences, Chongqing Medical University, Chongqing 400016, China

## Abstract

Molecular signatures are usually sets of biomolecules that can serve as diagnostic, prognostic, predictive, or therapeutic markers for a specific disease. Omics data derived from various high-throughput molecular biology technologies offer global, unbiased and appropriately comparable data, which can be used to identify such molecular signatures. To address the need for comprehensive disease signatures, DiSignAtlas (http://www.inbirg.com/disignatlas/) was developed to provide transcriptomics-based signatures for a wide range of diseases. A total of 181 434 transcriptome profiles were manually curated from studies involving 1836 nonredundant disease types in humans and mice. Then, 10 306 comparison datasets comprising both disease and control samples, including 328 single-cell RNA sequencing datasets, were established. Furthermore, a total of 3 775 317 differentially expressed genes in humans and 1 723 674 in mice were identified as disease signatures by analysing transcriptome profiles using commonly used pipelines. In addition to providing multiple methods for the retrieval of disease signatures, DiSignAtlas provides downstream functional enrichment analysis, cell type analysis and signature correlation analysis between diseases or species when available. Moreover, multiple analytical and comparison tools for disease signatures are available. DiSignAtlas is expected to become a valuable resource for both bioscientists and bioinformaticians engaged in translational research.

## Graphical Abstract

**Graphical Abstract:**
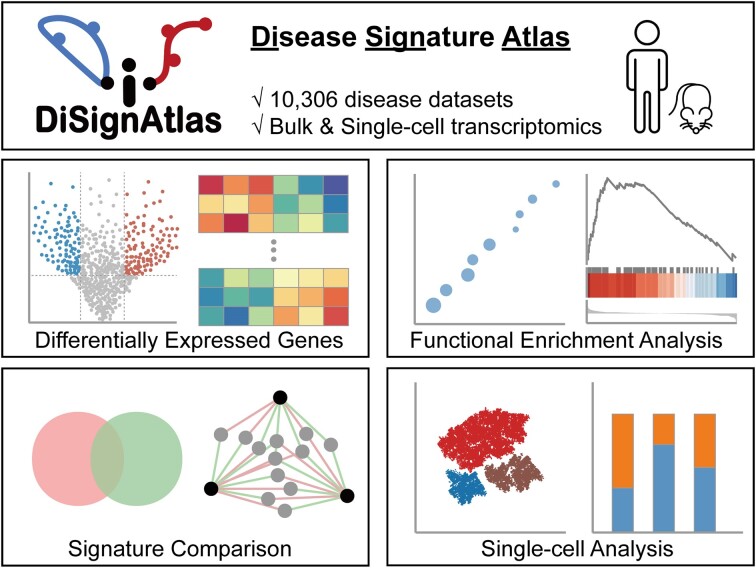

## Introduction

The rapid diagnosis and precise treatment of diseases, as well as the elucidation of disease pathogenesis, are among the major challenges in modern medicine. The discovery of disease-associated molecules, e.g. genetic variants, mRNAs, proteins, metabolites or other factors, plays a crucial role in addressing these challenges (1). These molecules can serve as valuable diagnostic, prognostic, predictive and therapeutic markers for specific diseases. Consequently, numerous studies have been conducted to identify and characterize disease-associated molecules. Molecular signatures, which usually encompass sets of biological molecules, are instrumental in disease research. Importantly, such signatures can be derived from diverse omics data types, allowing a more comprehensive, unbiased and comparable approach to identifying relevant molecular patterns (2). High-throughput sequencing technologies, including microarray, RNA-Seq, single-cell RNA-Seq (scRNA-Seq) and single-nucleus RNA-Seq (snRNA-Seq), enable the quantitative evaluation of gene expression to identify signatures of differential gene expression in diseases.

In recent decades, the burgeoning field of omics data analysis has given researchers the ability to explore and compare vast datasets, thereby uncovering crucial molecular signatures associated with diseases. A vast number of studies have reported high-throughput transcriptomic data collected from various diseases, including cancers, and the raw data generated are deposited in databases such as Gene Expression Omnibus (GEO) (3) and The Cancer Genome Atlas (TCGA) (4). Although some databases, such as Gene Expression Profiling Interactive Analysis (GEPIA) (5) and BioXpress (6), include archives of differentially expressed genes (DEGs) associated with cancers, to our knowledge, there is currently no database that contains comprehensive omics-based signatures for various diseases other than cancers. The absence of a centralized data portal for disease signatures poses obstacles to exploring, cross-evaluating and comparing biomarkers across different diseases. This limitation has also hindered data mining efforts and made it difficult for researchers to gain a comprehensive understanding of the molecular signatures associated with diverse diseases.

In this study, raw data representing 181434 unique transcriptome profiles of disease and control tissues from humans and mice were manually curated from GEO, TCGA and ArrayExpress (3,4,7). A total of 10 306 datasets related to 1836 nonredundant disease types, encompassing both disease and control groups, were meticulously organized and annotated. Among these datasets, 328 were derived from scRNA-Seq/snRNA-Seq experiments, offering single-cell comparisons. For each dataset, the raw data were reviewed and analysed systematically using standard pipelines to identify DEGs in various diseases. After this processing, 5498991 DEGs were identified and deposited as signatures of human and mouse diseases in the user-friendly database DiSignAtlas. This database provides downstream functional enrichment analysis, cell type analysis and improved, interactive data visualization. Therefore, DiSignAtlas can aid in identifying commonalities and disparities in gene expression patterns, pathways and cellular responses among different disease types. In brief, DiSignAtlas offers the most comprehensive atlas available of human and mouse disease signatures based on bulk and single-cell transcriptomics to link diseases with DEGs, pathways, cell types and tissues to interrogate the mechanisms, diagnosis and treatment of diseases as well as gene functions and disease models. We hope that this database will become an invaluable data resource for researchers in their pursuit of improved diagnostics, treatments and overall understanding of diseases.

## Materials and methods

### DiSignAtlas workflow

**Figure 1.**
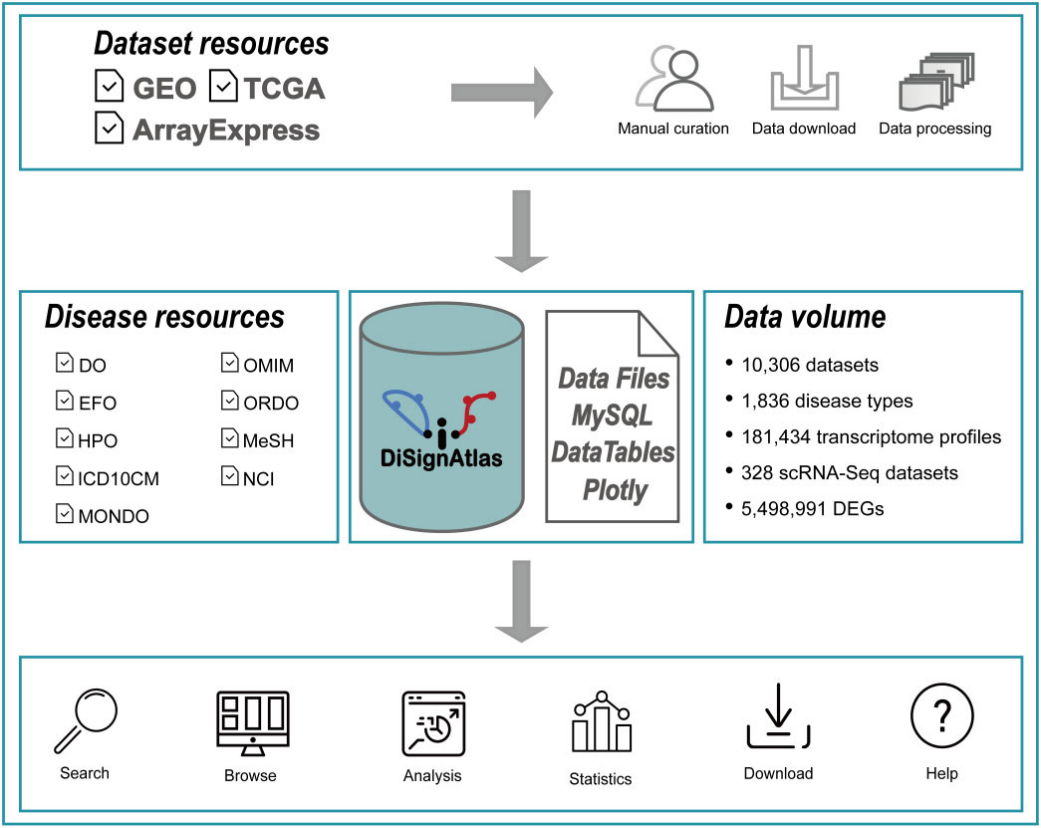
DiSignAtlas workflow. First, disease-related transcriptomics data were meticulously gathered and curated from publicly available repositories such as GEO, ArrayExpress and TCGA. These datasets were subsequently organized based on specific diseases and subjected to differential gene expression analysis and downstream functional enrichment analysis. The results are stored in a user-friendly database, offering users various options to explore the results through well-structured tables and visually informative graphs.

The workflow of DiSignAtlas 1.0 is illustrated in Figure 1. The methodology for constructing the database and guidelines for utilizing the web server are detailed below.

### Data collection and processing

We conducted manual searches in the GEO, ArrayExpress and TCGA (3,4,7) databases to identify human and mouse transcriptomics datasets related to specific diseases using disease-relevant keywords. Subsequently, we ensured that each dataset included both disease and control samples. Samples from a given study were categorized into ‘disease’ and ‘control’ groups and organized into a disease dataset with a disease type of interest. For each disease-control dataset, disease samples should carry the disease of interest, while controls should be independent of this disease. Although in most cases, controls represent normal conditions, they need not be in good health. If cases carry other disease(s), so do controls. Moreover, controls can carry the same disease as the disease group but of another grade/severity. In this case, the disease of interest could be labelled as ‘progressive disease’. The disease type of interest is a key feature of the dataset, while the disease all the samples carry is annotated as ‘characteristic’ of the dataset. This entire process was carefully validated by at least two researchers. Furthermore, we downloaded raw data and metadata for a significant proportion of the datasets, including expression profiles annotated with probe IDs, raw RNA-sequencing data and raw count data. For microarray data, we utilized the R packages GEOquery (8) and ArrayExpress (9) to download expression profiles from the GEO and ArrayExpress (3,7) databases, respectively. For RNA-Seq data, we primarily downloaded compressed FASTQ files from the European Nucleotide Archive (ENA) (10) and manually acquired processed count files from UCSC Xena.

Customized workflows were adopted for studies using different library construction strategies and are described in our previous studies (11,12). For microarray data, we utilized limma (v3.52.1) (13) to conduct differential expression analysis. In the case of bulk RNA-Seq data, we employed md5 checking and fastp quality control (v0.23.1) (14) and used HISAT2 (v2.2.1) (15), featureCounts (v2.0.1) (16), DESeq2 (v1.36.0) (17) or edgeR (v3.38.1) (18) for read alignment, estimation of gene expression levels and differential expression analysis of samples with or without duplicates, respectively. For scRNA-Seq data, we utilized SCTransform (v0.3.3) (19), ScType (20) and Seurat (v4.1.1) (21) to construct normalization matrices, annotate cell types and perform differential expression analysis, respectively. Default parameters were chosen for all the aforementioned analyses. The source codes and the command line parameters of the software used are provided on the download page of DiSignAtlas (http://www.inbirg.com/disignatlas/download). In each DiSignAtlas dataset, we identified DEGs through differential expression analysis using a significance threshold of adjusted *P*-value < 0.05; adjusted *P*-values were obtained from the Benjamini − Hochberg (BH) multiple testing correction method implemented in tools including limma, DESeq2 and edgeR (22). In instances where >1000 DEGs were identified, to better perform functional enrichment analysis and signature comparison analysis, we only selected the top 1000 DEGs based on absolute log_2_-fold change.

### Enrichment analyses

Enrichment analysis was performed on the DEGs that met the adjusted *P*-value threshold (23) for each DiSignAtlas dataset using the R package ClusterProfiler 4.0 (24). The analysis included regulatory transcription factor enrichment from TRRUST (version 2) (25), Gene Ontology (GO) term (26) and KEGG pathway (27) enrichment, and gene set enrichment analysis (GSEA). Additionally, we explored distinct cell type markers obtained from the CellMarker (28) and PanglaoDB (29) databases. The source codes can be downloaded on the DiSignAtlas website.

### Signature comparisons

Significance was assessed through hypergeometric tests when comparing signatures within the DiSignAtlas dataset and when comparing these signatures to user-submitted gene sets. In cases where lists for both up- and downregulated genes were available, the fold enrichment and *P*-values were averaged to derive the final value. Furthermore, the upregulated gene list in one signature was compared not only to the upregulated gene list in the other signature to test for a positive correlation but also to the downregulated gene list in the other signature to test for a negative correlation. The correlation with the smallest *P*-value was chosen as the ultimate result of the comparison. A significance threshold of *P*-value <0.05 was established to determine the significance of enrichment.

### Web interface implementation

The database is constructed on a Linux server utilizing Nginx (v1.14.1) as the web server software and uWSGI (v2.0.20) as the application server interface. Metadata and analysis results are stored in the MySQL (v8.0.26) database. Back-end data exchange is implemented using Django (v2.1.8), while front-end interactive visualization is achieved through the utilization of Bootstrap (v4.3.1), jQuery (v3.2.1), DataTable (v1.10.21), ECharts (v5.0.2) and Plotly (v2.8.3). Statistical analysis was performed using Python packages such as numpy (v1.21.2), Pandas (v1.3.5), GSEApy (v0.10.8) and SciPy (v1.7.3) (30).

## Results

### Database content and statistics

The latest DiSignAtlas release comprises an extensive collection of 181 434 bulk and single-cell transcriptome profiles. Within this database, there are 10 306 comparative datasets, including 328 scRNA-Seq/snRNA-Seq datasets, which have been meticulously organized and analysed. These datasets encompass a diverse range of diseases found in both humans and mice. A grand total of and 1836 distinct disease types were involved. Among these disease types, a staggering 5498991 DEGs associated with various diseases, such as Alzheimer's disease, COVID-19 and asthma, were successfully identified. Interactive graphs are provided on the statistics page. For example, 2 of the top 10 DEGs by frequency, i.e. *CXCL10* and *SPP1*, are shown in both human and mouse diseases. The histogram of the number of samples in the disease datasets indicates that most datasets have 5–9 samples. The Venn diagram shows that 261 disease types overlap between humans and mice.

### Web design and interface

**Figure 2.**
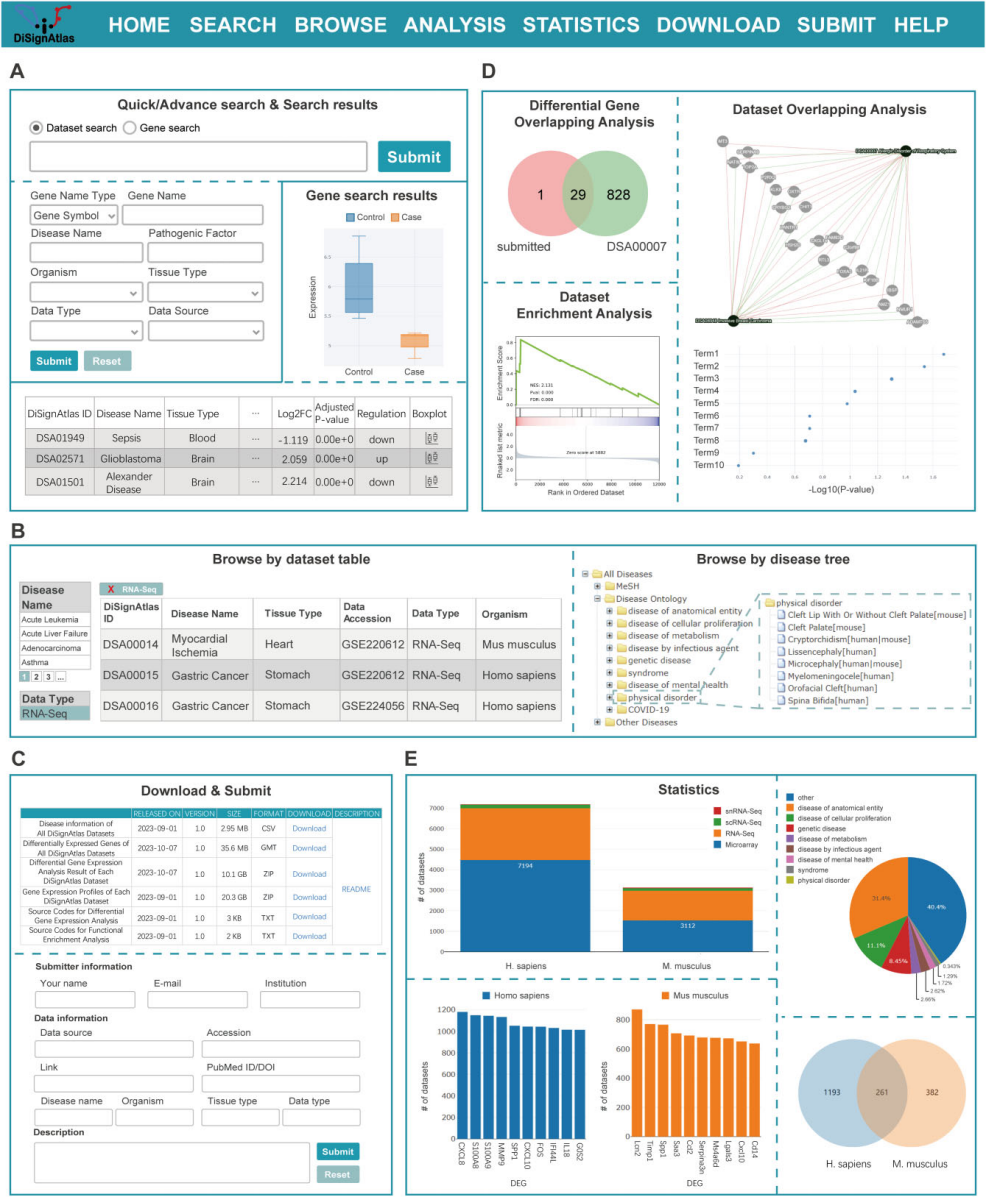
Contents of DiSignAtlas. (**A**) Search section: users can conduct quick searches using disease keywords or gene information directly from the home page. From the search page, users can perform more advanced searches by filtering one or more features of a disease dataset. Datasets meeting the criteria are listed in a table on the results page. (**B**) Browse section: users can browse datasets filtering by disease name, data type, tissue type and organism; users can also browse disease types in disease trees. (**C**) Download and submit section. (**D**) Analysis section, with three analysis tools: differential gene overlap analysis helps to study disease datasets that are significantly enriched in a user's gene set by hypergeometric tests; dataset enrichment analysis helps identify disease datasets in which a user's gene set is overrepresented; and dataset overlap analysis displays common functions and DEGs of found disease datasets in the results page. (**E**) Statistics section: statistical graphs are available to visualize the distribution of datasets, disease types and DEGs.

DiSignAtlas offers a multitude of data retrieval methodologies through its user-friendly web interface (Figure 2). There are four main data retrieval methods: Quick Search, Advanced Search, Browse and Download (Figure 2A–C). Upon visiting the detailed information page, the user will find a comprehensive overview of the disease dataset along with the analysis results. Furthermore, DiSignAtlas provides users with three practical analysis tools, namely, differential gene overlap analysis, dataset enrichment analysis and dataset overlap analysis (Figure 2D). The statistics page shows the details of the statistical outcomes derived from the dataset (Figure 2E). Additionally, the download page contains links to all datasets, the original data sources, DEGs, differential gene expression analysis results and gene expression profiles of each dataset, facilitating access to these resources (Figure 2C). Moreover, the source codes of differential gene expression analysis and functional enrichment analysis are provided on the download page as well. DiSignAtlas also warmly invites users to submit new disease data via the submission page (Figure 2C). If assistance is needed, the help page provides detailed step-by-step tutorials and contact information. The following section elaborates on some of these functionalities in greater detail.

#### Data searching

DiSignAtlas features two data searching methods: Quick Search and Advanced Search. These methods allow the efficient retrieval of disease datasets based on user preferences. On the home page, users can use Quick Search methods, including Disease Dataset Search and Differential Gene Search (Figure 2A). By selecting the ‘Search by Disease Dataset’ option, users can input a keyword related to the disease dataset they are interested in, such as the disease name (e.g. Alzheimer's disease), tissue (e.g. kidney), or data type (e.g. scRNA-Seq). Alternatively, by selecting the ‘Search by Differential Gene’ option, users can enter a gene name or ID and search for disease datasets associated with the submitted gene. Moreover, an Advanced Search option is available on the search page. With this option, users can filter disease datasets according to various features, including ‘gene’, ‘pathogenic factor’, ‘organism’, ‘tissue’, ‘disease name’, ‘disease type’ and ‘data type’. By applying specific criteria, disease datasets meeting the selected features are listed on the results page.

#### Data browsing

On the browse page of DiSignAtlas, users have the opportunity to view the available datasets and disease types in an organized manner (Figure 2B). One section presents the whole disease dataset listed in an interactive table, which supports further filtering using options such as disease name, data type, tissue type and organism. Another visually engaging feature is the disease tree. Two disease trees are provided: one contains all disease types in both humans and mice, and each disease type is annotated to the involved organism(s) in the database; the other contains common disease types between humans and mice. These trees provide a comprehensive view of all diseases categorized according to two taxonomies: Medical Subject Headings (MeSH) (31) and Disease Ontology (DO) (32). Diseases that are not present in either of these two databases are grouped under the category ‘Other Diseases’. This classification system helps users quickly locate diseases of interest within different taxonomic hierarchies. Additionally, the home page features a disease cloud, which visually represents the distribution and prominence of different diseases in the DiSignAtlas database. The size of each disease name in the cloud is proportional to its representation in the datasets, offering a quick glimpse of the representation of different diseases in the platform.

#### Detailed information

**Figure 3.**
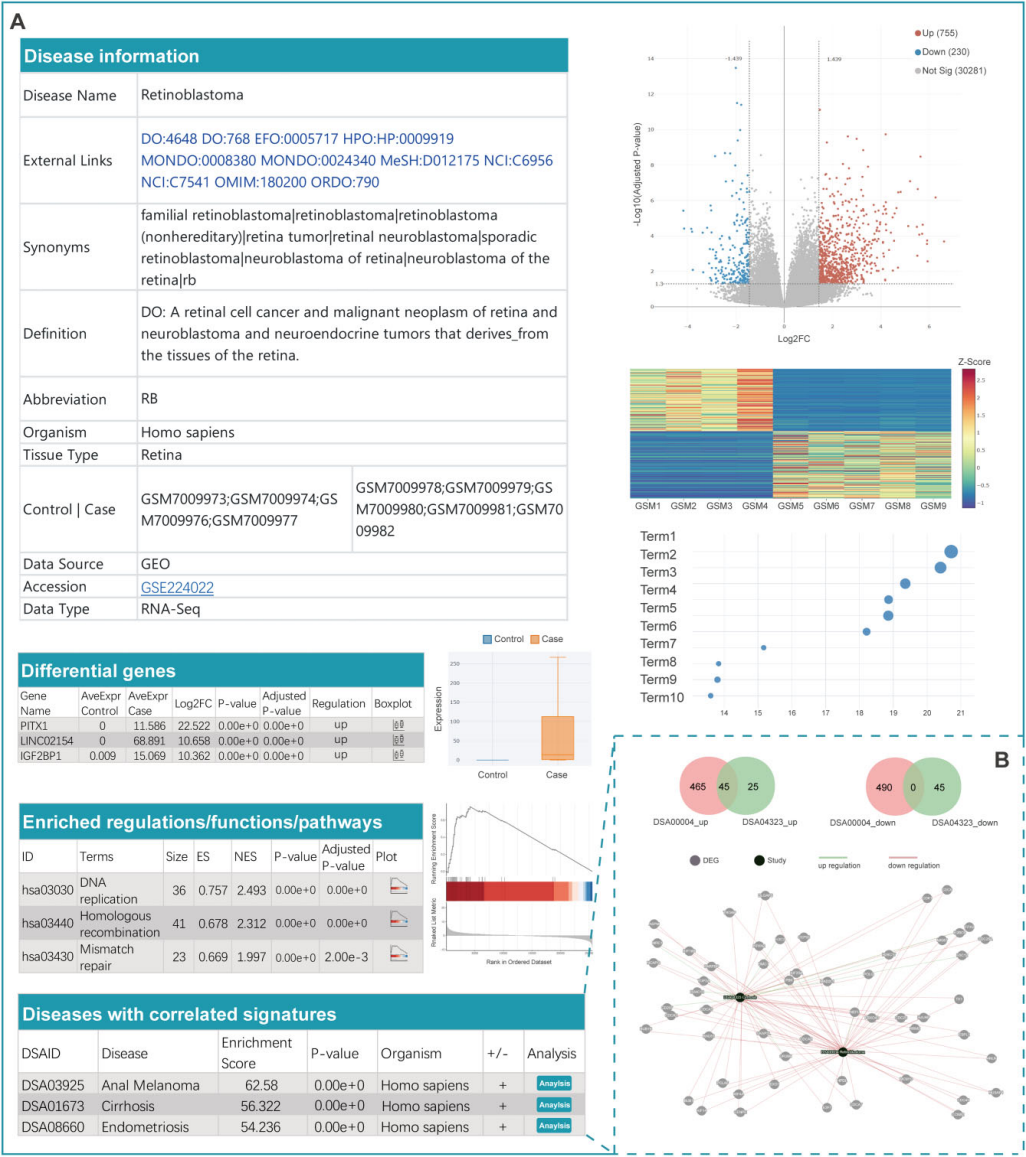
Detailed information for bulk datasets. (**A**) Various essential components of the disease dataset are shown, including disease information, disease definition, differential genes, enriched functions/pathways and diseases with correlated signatures. (**B**) Clicking on the ‘Analysis’ button leads to a page that comprehensively illustrates the overlapping DEGs and functional enrichment results.

**Figure 4.**
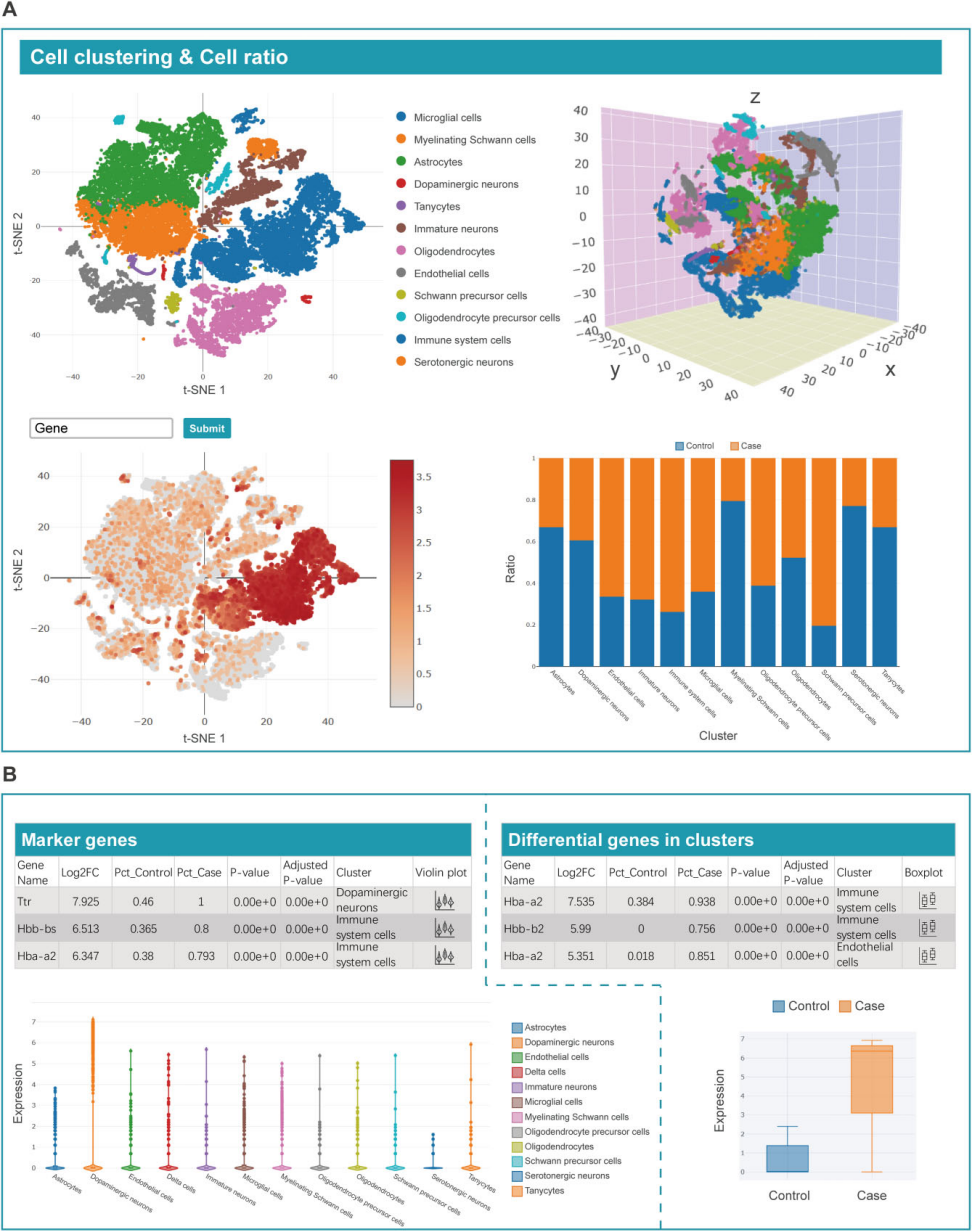
Detailed information for single-cell datasets. (**A**) Cluster analysis of single-cell data. (**B**) Visualization of marker genes and differentially expressed genes in distinct cell populations.

When a user clicks on a DiSignAtlas ID on the Results or browse page, they are redirected to the corresponding dataset's details page (Figure 3). This details page provides comprehensive information related to the disease dataset, including various key elements. Under the Disease Information section, users can find essential details about the disease, such as its name, definition, involved tissue types, relevant references and external links to other databases such as DO, Experimental Factor Ontology (EFO), Human Phenotype Ontology (HPO), Monarch Disease Ontology (MONDO), MeSH, Orphanet Rare Disease Ontology (ORDO), Online Mendelian Inheritance in Man (OMIM) and National Cancer Institute (NCI) (31–38) (Figure 3A). The Differential Genes section presents a list of DEGs identified as associated with a given disease. These DEGs are illustrated through boxplots in the table. Additionally, volcano plots and heatmaps are provided to visually represent the differential gene expression patterns. Users can also explore differential cell type markers associated with the disease. For further insight into disease-related functions and pathways, the details page includes information about the enrichment of upstream transcription factors and downstream functions/pathways. This enhances the user's understanding of the molecular mechanisms underlying the disease. In the case of diseases with correlated features, DiSignAtlas offers overlap analysis using the hypergeometric test, which provides information about diseases with positively or negatively correlated characteristics (Figure 3A, B). Moreover, the details page provides tools for single-cell clustering analyses (Figure 4). Users can explore cell cluster maps, cell ratios, cluster marker genes and DEGs in clusters at different resolutions and with different types of visualizations (t-SNE or UMAP) (Figure 4A, B). If available, cell types can also be inferred from the analysis. The cell ratio plot visually displays the proportion of cells in each cluster for the disease and control groups, providing valuable insights into cell population changes.

### Analytical functions

DiSignAtlas offers three practical analysis functions: differential gene overlap analysis, dataset enrichment analysis and dataset overlap analysis. These tools empower users to compare their genetic signatures with those in the DiSignAtlas datasets or perform comparisons between retrieved DiSignAtlas datasets (Figure 2).

*Differential gene overlap* analysis allows users to discover disease datasets in DiSignAtlas whose DEGs significantly overlap with a user-submitted gene set. Users can input a list of DEGs, specifying whether they are up- or downregulated. The analysis then maps these genes to relevant datasets from the same organism (human or mouse) and performs hypergeometric tests to identify datasets with significant enrichment. The eligible datasets are sorted by *P*-value, enabling users to identify the most significantly relevant disease datasets. Additionally, significant enrichment in datasets from another organism can be obtained by transforming the submitted gene list to orthologous genes and then mapping them to the relevant studies. Moreover, clicking on the dataset ID brings up a new page containing the Results table, a Venn diagram and a display of overlapping genes between two traits.

*Dataset enrichment analysis* helps to identify disease datasets in DiSignAtlas that are overrepresented in a user-submitted gene set. Users furnish a set of genes, and the analysis proceeds to execute the GSEA algorithm across all DiSignAtlas datasets for the specified organism. Additionally, it considers homologous genes present in datasets from another organism. The enriched datasets are returned and sorted by enrichment score, allowing users to identify datasets with high relevance to the submitted gene set.

*Dataset overlap analysis* enables users to perform comparisons between different datasets in DiSignAtlas. When multiple related datasets are found, the dataset overlap analysis is displayed on the results page or in the ‘Diseases with correlated signatures’ section of the details page. The analysis displays overlapping downstream DEGs of diseases in a network format, helping users identify shared genetic features. Additionally, the GO terms or KEGG pathways associated with DEGs according to functional enrichment analysis are listed, providing insights into the biological functions and processes implicated in the disease datasets.

### Case study

To demonstrate the application of DiSignAtlas, we investigated influenza, commonly known as ‘the flu’, as an example. Influenza is an infectious disease caused by influenza viruses that infect the nose, throat and sometimes the lungs. Using the advanced search, we can search ‘influenza’ in the disease form and ‘Homo sapiens’ in the organism form to yield 118 influenza-related datasets (Supplementary Table S1). Clicking the ‘overlapping analysis’ button at the bottom of the results page shows the common DEGs of those datasets. Eighteen genes in at least eight datasets were dysregulated (Supplementary Table S2). Among those genes, *IFI27*, *IFI35*, *IFI44L*, *IFIT1*, *IFIT3* and *IFITM3* belong to the interferon alpha gene family. All of them have been identified as promising biomarkers and/or therapeutic targets for influenza infection (39–42). Then, we further examined one of the datasets, ‘DSA08144’. The detailed information page shows that ‘viral process’ was enriched in the functional analysis of DEGs in influenza. In the section ‘Diseases with correlated signatures’, 9 and 2 of the top 10 human and mouse disease datasets sorted by enrichment score are influenza-related datasets with positively correlated signatures, respectively (Supplementary Table S3). This indicates that mice with influenza share similar signatures with humans and can therefore be used as disease models for studies of human influenza. The search results indicate that DiSignAtlas could help to explore the diagnosis, mechanism and models of human diseases.

## Conclusions and future directions

Molecular signatures, including genetic variants, proteins and metabolites, play a crucial role in biomedical research. These signatures can be employed in various domains, such as disease diagnosis, prognosis, disease progression and personalized medicine. Identifying the genetic signatures associated with disease perturbations is essential for studying gene functions, biological processes and mechanisms of diseases. While numerous studies report disease–gene associations, comparing the associations systematically is often challenging due to differences in methods employed and the frequent focus on individual genes. Omics-derived signatures can address this challenge effectively. Over the years, a substantial amount of transcriptomic data have been generated from various disease contexts in humans and mice. However, these data are often disorganized and scattered, hindering knowledge mining. In contrast, the data in DiSignAtlas have been meticulously gathered and processed using standardized methodologies, ensuring that they can be readily compared and analysed, with 10 306 organized datasets in the current version. DiSignAtlas offers the ability to examine alterations caused by disease perturbations at multiple levels, including the gene, pathway and cell type levels. DiSignAtlas stands out with three novel features. First, it possesses the largest number of datasets of any database of its kind, not only for cancer but also for various types of diseases in both humans and mice. Second, signatures derived from transcriptomic data enable comparisons between diseases or even between human and mouse diseases. Third, DiSignAtlas includes 328 scRNA-Seq datasets, allowing single-cell analysis to be performed. We plan to regularly update DiSignAtlas every 3 months with newly released data, and we further intend to incorporate other omics data, such as genomics and proteomics data, in the future. Additionally, future versions will include data from diseases in other organisms. We will also develop and incorporate additional analytical tools into DiSignAtlas to compare and integrate additional aspects of the disease datasets.

In summary, DiSignAtlas serves as a comprehensive resource for transcriptomics-based signatures related to various diseases. It not only provides links between diseases and DEGs, pathways and cell types but also enables comparisons among diseases and organisms. As the most comprehensive database of omics-based disease signatures currently available, DiSignAtlas is expected to facilitate the exploration of diagnostic markers, disease models, mechanisms of action and treatment approaches for various diseases. DiSignAtlas is expected to be an invaluable resource for bioscientists and bioinformaticians conducting translational research.

## Supplementary Material

gkad961_supplemental_files

## Data Availability

DiSignAtlas is freely available online at http://www.inbirg.com/disignatlas/, and there is no login requirement. The analysis results and source codes are available on the DiSignAtlas website.
