# Supplementary material for: DiSignAtlas: an atlas of human and mouse disease signatures based on bulk and single-cell transcriptomics
Source: Nucleic Acids Res. 2023 Nov 1;52(D1):D1236–45. doi: 10.1093/nar/gkad961 (PMC10767933; doi:10.1093/nar/gkad961)
Supplement: gkad961_supplemental_files [file gkad961_supplemental_files.zip › supplemental_file.pdf]

Supplementary information

DiSignAtlas: an atlas of human and mouse disease signatures based on bulk and single-cell transcriptomics

---

Supplementary Table S1. 118 DiSignAtlas datasets found by searching 'influenza'

Supplementary Table S2. Genes that were dysregulated in at least 2 influenza-related datasets

Supplementary Table S3. Top 10 human and mouse disease datasets with correlated signatures sorted by enrichment score compared to dataset 'DSA08144'
